# Distinct calcium regulation of TRPM7 mechanosensitive channels at plasma membrane microdomains visualized by FRET-based single cell imaging

**DOI:** 10.1038/s41598-021-97326-z

**Published:** 2021-09-09

**Authors:** Irina Starostina, Yoon-Kwan Jang, Heon-Su Kim, Jung-Soo Suh, Sang-Hyun Ahn, Gyu-Ho Choi, Myungeun Suk, Tae-Jin Kim

**Affiliations:** 1 Department of Integrated Biological Science, Pusan National University, Pusan, 46241 Republic of Korea; 2 Department of Biological Sciences, Pusan National University, Pusan, 46241 Republic of Korea; 3 Department of Mechanical Engineering, Dong-Eui University, Pusan, 47340 Republic of Korea

**Keywords:** Fluorescence imaging, Fluorescent proteins, Fluorescence resonance energy transfer

## Abstract

Transient receptor potential subfamily M member 7 (TRPM7), a mechanosensitive Ca^2+^ channel, plays a crucial role in intracellular Ca^2+^ homeostasis. However, it is currently unclear how cell mechanical cues control TRPM7 activity and its associated Ca^2+^ influx at plasma membrane microdomains. Using two different types of Ca^2+^ biosensors (Lyn-D3cpv and Kras-D3cpv) based on fluorescence resonance energy transfer, we investigate how Ca^2+^ influx generated by the TRPM7-specific agonist naltriben is mediated at the detergent-resistant membrane (DRM) and non-DRM regions. This study reveals that TRPM7-induced Ca^2+^ influx mainly occurs at the DRM, and chemically induced mechanical perturbations in the cell mechanosensitive apparatus substantially reduce Ca^2+^ influx through TRPM7, preferably located at the DRM. Such perturbations include the disintegration of lipid rafts, microtubules, or actomyosin filaments; the alteration of actomyosin contractility; and the inhibition of focal adhesion and Src kinases. These results suggest that the mechanical membrane environment contributes to the TRPM7 function and activity. Thus, this study provides a fundamental understanding of how the mechanical aspects of the cell membrane regulate the function of mechanosensitive channels.

## Introduction

Transient receptor potential subfamily M member 7 (TRPM7) not only mediates the transport of Ca^2+^, Mg^2+^, and Zn^2+^ ions through the cell plasma membrane (PM)^1^, thereby supporting the homeostasis of these divalent ions in cells, but also has a C-terminal serine/threonine kinase domain that phosphorylates various downstream substrate molecules. TRPM7 is expressed ubiquitously and plays an important role in vital cell functions, as well as in the pathophysiology of cancer and cardiovascular and neurodegenerative diseases^2^. TRPM7 is locally associated with the focal adhesion (FA) complex and participates in the regulation of actomyosin contractility and cell interactions with the extracellular matrix^3,4^, but it remains unclear how these and other mechanical cues of a cell affect TRPM7 activity.

Lateral organization of the cell PM into domains with different properties is suggested by the lipid rafts hypothesis, wherein lipid rafts are microdomains of the cell PM that are rich in cholesterol and sphingolipids^5^. The PM in living cells is intricately arranged, and detergent-resistant membranes (DRMs) obtained through destructive methods, such as cold-temperature detergent solubilization, cannot represent lipid rafts in vivo^5^. For convenience, the terms “lipid raft” and “DRM” are equated in this study. Lipid rafts are directly mechanosensitive and are rapidly organized around new or deformed cell-extracellular matrix contact^6^. Lipid rafts regulate the actin cytoskeleton and FA dynamics; after lipid raft disruption, actin stress fiber (SF) formation is enhanced and FA disassembly is inhibited^7^. Lipid rafts are highly ordered in FAs but quickly lose their order after cell detachment from the extracellular matrix^8^.

Another essential structure of non-muscle cell is the SF network. Each SF comprises 10–30 actin filaments that are cross-linked with α-actinin. Actin filaments are linear polymers of actin monomers that can associate with non-muscle myosin II, which together likely act as a contractile motor^9^. SFs are often attached to FAs via intermediate proteins, thus making SFs a part of the mechanosensitive apparatus of cells^9^ and allowing them to participate in cell adhesion, migration, and morphogenesis^10^. Myosin light chain kinase (MLCK) can directly phosphorylate myosin II regulatory light chain, making MLCK an important regulator of the actomyosin contractility chain^11,12^. Another regulator is RhoA from a family of small GTPases. GTP binds to RhoA, which then activates Rho-associated coiled-coil forming kinase (ROCK)^13^. ROCK phosphorylates the non-muscular myosin II regulatory light chain, which activates myosin. The myosin light chain phosphatase (this phosphatase, once phosphorylated, is unable to dephosphorylate the regulatory chain of the non-muscular myosin II^14^); and the mammalian homolog of *Drosophila diaphanous* mDia, which is involved in the polymerization of actin SFs, enhance the protrusive forces^15^.

In addition to SFs, microtubules are important components of the cytoskeleton. Microtubules target their growth toward FAs using actin filaments as a guide. Microtubules stimulate FA turnover by acting as rails for the delivery of exocytic vesicles that can disassemble FAs. Additionally, microtubules can regulate the Rho family member Rac, affecting actin filament growth and indirectly influencing actin dynamics and actomyosin contractility^16^. FAs are the sites of integrin clustering in the cell PM. They contain multiple proteins that provide scaffolding and signaling features, forming mechanical links between the extracellular matrix and SFs. FA kinase (FAK) serves as a mechanical adaptor in FAs and is an intermediate component in signaling pathways. Src kinase binds to FAK, thereby triggering its phosphorylation and activation. Src kinase also binds to growth factor receptors. Thus, FAK can directly conduct signals from both integrins and growth factor receptors via Src downstream to the Ras-mitogen-activated protein kinase cascade. This is important for the regulation of adhesion dynamics, cell migration, cell survival, cell growth^17^, and FA turnover^18^.

The aim of this study was to reveal whether the above-described structures are involved in the regulation of the TRPM7 channel. Thus, the normal functions of these structures were altered using chemicals or by transfecting mutant constructs into cells. Live-cell imaging of cells transfected with biosensors that reported Ca^2+^ concentration changes was conducted. For the activation of TRPM7, its specific agonist naltriben was used. The results are expected to determine whether mechanical cues of a cell regulate TRPM7 activity and thus lay a foundation for the understanding of transmembrane channel mechanical regulation.

## Materials and methods

### Cell culture

Breast cancer Michigan Cancer Foundation-7 (MCF-7; Korean Cell Line Bank, Seoul, Republic of Korea) cells were used for all experiments. Dulbecco’s modified Eagle’s medium (DMEM; CM002, GenDEPOT, Katy, TX, USA) supplemented with 10% (v/v) fetal bovine serum (FBS; WB0015, HyClone, Logan, UT, USA) and 1% (v/v) penicillin–streptomycin (P/S) solution (100X; 100 units/mL of penicillin and 100 μg/mL of streptomycin; CA005, GenDEPOT, Katy, TX, USA) was used for cell maintenance. Cells were incubated at 37 °C in humidified conditions with 95% air and 5% CO_2_.

### DNA plasmid transfection

The construction of DNA plasmids encoding Ca^2+^-sensitive biosensors was undertaken following previously described methods^19^. D3cpv was gifted by Dr. Yingxiao Wang (University of California, San Diego, CA, USA), and RhoA mutants were gifted by Dr. Jihye Seong (Korea Institute of Science and Technology, Republic of Korea). Before and after plasmid transfection with Lipofectamine 3000 (Invitrogen, Carlsbad, CA, USA), MCF-7 cells were kept in confocal dishes (Cat. No. 100350, SPL Life Sciences, Pocheon, Republic of Korea) with DMEM (10% FBS and 1% P/S) in a humidified incubator for at least 24 h. For co-transfection, plasmids were used at a 1:1 ratio.

### Chemicals and reagents

Starvation medium (DMEM with 0.5% FBS and 1% P/S) was applied to transfected cells for 4–12 h before live-cell imaging, for which the medium was changed to CO_2_-independent medium (Cat no. 18045088, Gibco, Waltham, MA, USA) with 0.5% (v/v) FBS and 4 mM of L-glutamine. Next, if required by the conditions of the experiment, the cells were pretreated with chemicals dissolved in the CO_2_-independent medium. For this, methyl-beta-cyclodextrin (MBCD; Sigma Aldrich, St. Louis, MO, USA; 5 mM for 1 h), naltriben (50–100 μM), ML-7 (Sigma Aldrich; 10 μM for 1 h), cytochalasin D (ab143484, Abcam, UK; 1 μM for 30 min), nocodazole (S2013, Selleck Chemicals, Huston, TX, USA; 5 μM for 1 h), PF-573228 (S2013, Selleck Chemicals; 5 μM for 1 h), or PP1 (14,244, Cayman chemical, Ann Arbor, MI, USA; 10 μM for 1 h) were used. Thapsigargin (T9033; 10 μM for 30 min) is commercially available from Sigma-Aldrich. For extracellular calcium-free condition, Ca^2+^ free Hanks balanced salt solution (HBSS) containing 20 mM HEPES, 0.5 mM EGTA, 1 mM MgCl_2_, and 1 mM MgSO_4_ (pH 7.4) was used.

### Fluorescence resonance energy transfer (FRET)-based live cell imaging

Images were acquired with the charge-coupled device camera of the DMi8 microscope (DFC450C, Leica, Germany). A 436/20 nm excitation filter, 455 nm dichroic mirror, and 535/30 nm for the “FRET” channel and 480/40 nm for the “enhanced cyan fluorescent protein” channel (named as "CFP" in Figures here) emission filter was set. Using Leica LAS X 3.6.0. software (Leica; https://www.leicamicrosystems.com/products/microscope-software/p/leica-las-x-ls/), the background of each channel was removed, and the pixel-by-pixel ratio of the “FRET” channel to the “CFP” channel in the regions of interest within the cell images was calculated.

### Immunofluorescence (IF) staining and imaging

MCF-7 cells were used for IF staining. First, cells were fixed with 4% paraformaldehyde in phosphate-buffered saline (PBS) for 10 min, then washed three times with PBS for 10 min (this washing occurred prior to each following step). After permeabilization with Triton X-100 (Sigma, Cat. No. STBG3972V) in PBS for 15 min, cells were blocked with 3% bovine serum albumin. Staining with the primary antibody mouse anti-TRPM7 (1:200, GeneTex, GTX41997) was conducted overnight at 4 °C, and secondary antibody fluorescein isothiocyanate (FITC)-conjugated anti-mouse immunoglobulin G (1:200, Santa Cruz, sc-516140) was applied for 2 h. After 10 min, 4′,6-diamidino-2-phenylindole (DAPI) and Hoechst (33342, Invitrogen) staining samples were treated with mounting buffer (90% glycerol, 10% PBS, and 5 mM n-propyl gallate). Images were obtained with the same Leica DMi8 microscope with a 350/25 nm excitation filter, 400 nm dichroic mirror, and 460/25 nm emission filter for DAPI and a 480/20 nm excitation filter, 505 nm dichroic mirror, and 527/15 nm emission filter for FITC.

### Statistical analysis

GraphPad Prism 7.0.0 (GraphPad Software, La Jolla, CA, USA; https://www.graphpad.com/) was used for the statistical evaluation of data. Unpaired *t* test and one-way analysis of variance were used for intergroup difference determination. All results were expressed as mean ± standard error of the mean, and a *P* value < 0.05 was considered significant.

## Results

### TRPM7-induced Ca^2+^ influx mainly occurs at the DRM domains

**Figure 1:**
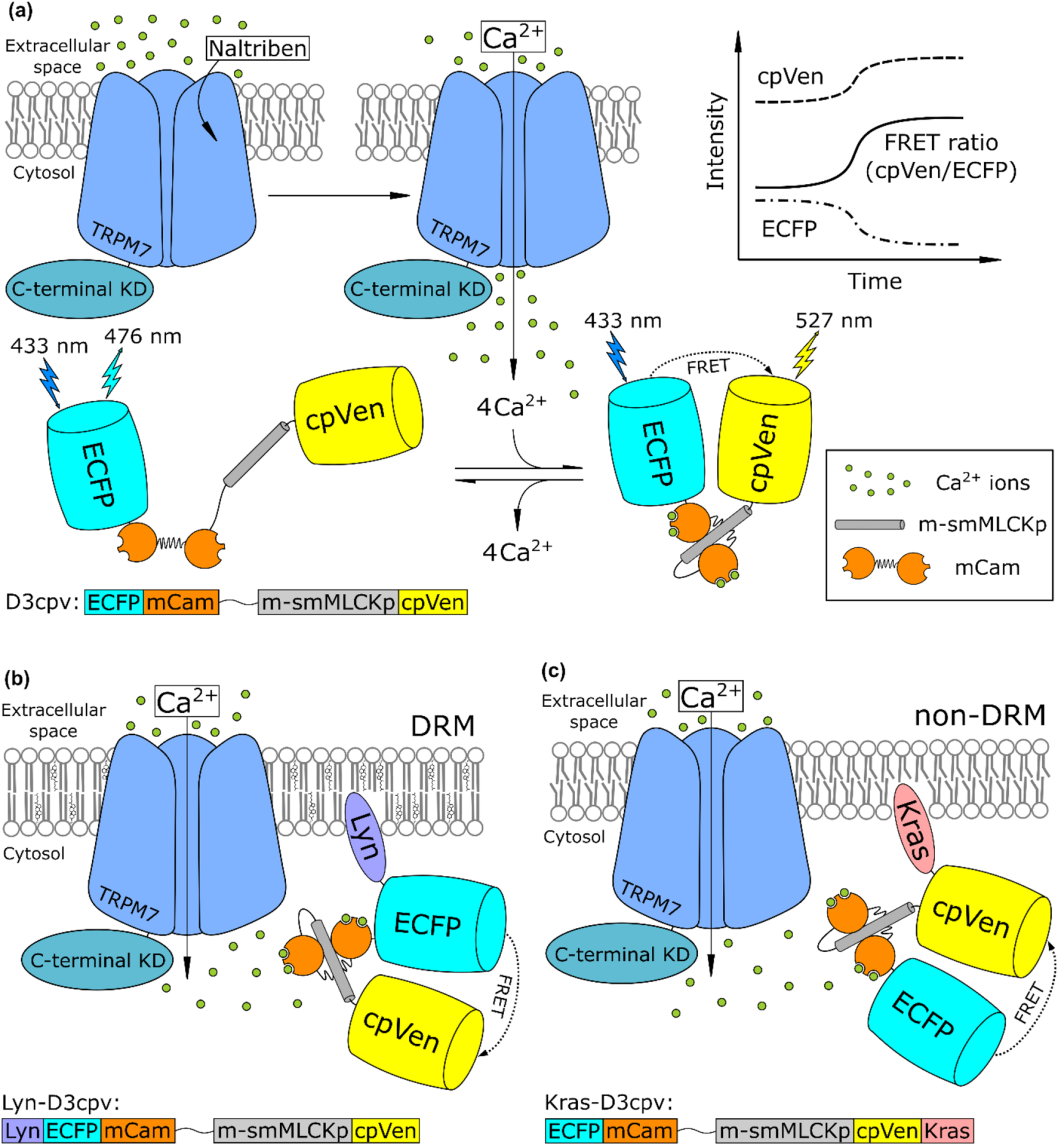
Schematic representation of the experiment. The local Ca^2+^ concentration increased after transient receptor potential subfamily M member 7 activation with naltriben and (**a**) the cameleon biosensor D3cpv changed its conformation in the presence of Ca^2+^ ions resulting in fluorescence resonance energy transfer (FRET) ratio change. Conformational change occurs due to Ca^2+^ binding to mutated calmodulin (mCam) and consequent interaction of this complex with mutated smooth muscle myosin light chain kinase peptide (m-smMLCKp). (**b**) Lyn-D3cpv contains the lyn kinase acylation substrate sequence at the N-terminus, (c) and a prenylation substrate sequence from KRas is fused with the C terminus in Kras-D3cpv, which causes tethering of the biosensors to detergent-resistant membrane (DRM) and non-DRM. Figures from several studies were used as a reference for the preparation of this figure^19,25–31^. KD, kinase domain.

**Figure 2:**
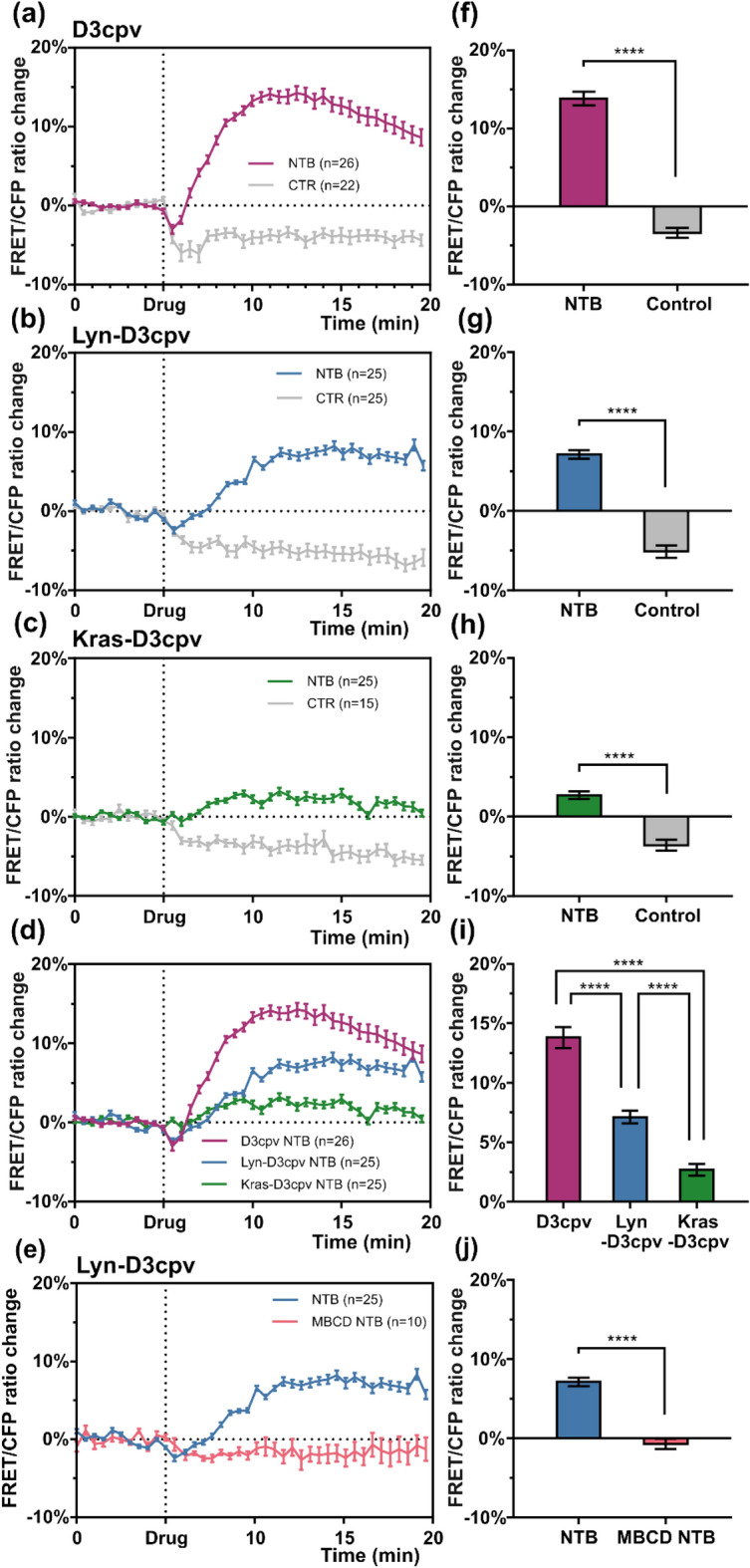
Graphs of the fluorescence resonance energy transfer (FRET)/enhanced cyan fluorescent protein (CFP) ratio change showing the difference in transient receptor potential subfamily M member 7 activity at distinct microdomains of a cell PM. The results were obtained from the following biosensors: (**a**) D3cpv, (**b**) Lyn-D3cpv, and (**c**) Kras-D3cpv. (**d**) The superposition of line graphs from (**a**), (**b**), and (**c**). (**e**) The FRET/CFP ratio change of Lyn-D3cpv from cells pretreated with methyl-beta-cyclodextrin compared to those that were untreated. Bar plots with the values of the FRET/CFP ratio change 12 min after the start of experiment (**f**) for the data from (**a**), (**g**) from (**b**), (**h**) from (**c**), and (**j**) from (**e**). Student’s *t* test, ****P < 0.0001. (**i**) Bar plots with the values from (**d**) 12 min after the start of the experiment. One-way analysis of variance, ****P < 0.0001. NTB, naltriben; n, number of the cells; CTR, control (dimethyl sulfoxide). Error bars in the line plots represent the standard error of the mean (*P* < 0.05).

**Figure 3:**
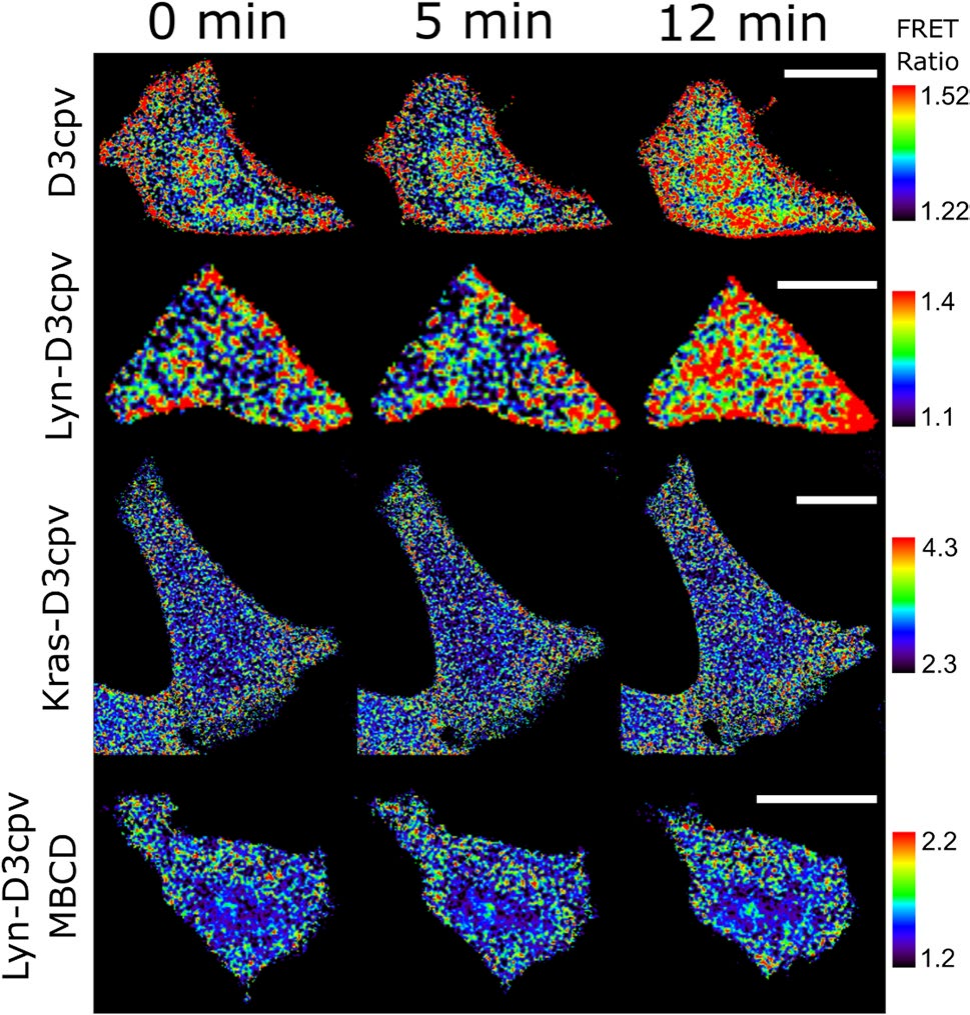
Time-lapse fluorescence resonance energy transfer (FRET) ratio images of the MCF-7 cells in false colors. Naltriben was added 5 min after the beginning of the experiment. The colored scale bar represents the range of the biosensor FRET/enhanced cyan fluorescent protein (CFP) emission ratios. The warmer the color of the pixel, the higher the Ca^2+^ concentration. Scale bar = 20 μM.

To determine the TRPM7 channel activity in distinct domains of the cell PM, MCF-7 cells were treated with the selective TRPM7 activator naltriben^20^. Due to this activation, Ca^2+^ concentration increased close to the TRPM7 channels. Transfected Lyn-D3cpv and Kras D3cpv allowed the monitoring of this concentration change at the DRM and non-DRM domains of the PM, respectively (Fig. 1)^19^. As shown in Figs. 2 and 3, the increase in the FRET ratio signal level from Lyn-D3cpv was much higher than that from Kras-D3cpv, indicating that TRPM7 could be located mainly at the DRM domains of the PM. To confirm whether the increase in FRET signal following naltriben treatment is caused by Ca^2+^ influx through TRPM7 channels, we examined it after blocking ER Ca^2+^ ATPase pump under extracellular Ca^2+^ free conditions. As a result, we observed that naltriben treatment did not trigger Ca^2+^ influx in Lyn-D3cpv transfected cells under these conditions, indicating TRPM7-specific Ca^2+^ signal (Supplementary Fig. 1).

### TRPM7-induced Ca^2+^ influx ceases after the disruption of DRM

To determine how the destruction of lipid rafts affects the activity of TRPM7, pretreatment of MCF-7 cells with MβCD was carried out. Lipid rafts are enriched in cholesterol. MβCD captures cholesterol from lipid rafts, causing lipid rafts structure disintegration^21^. During subsequent live-cell imaging performed with transfected Lyn-D3cpv in MCF-7 cells, TRPM7-induced Ca^2+^ influx in cells pretreated with MβCD was greatly diminished compared to that in cells without pretreatment (Figs. 2, 3). These results suggest that the presence of lipid rafts is necessary for the activation of TRPM7 channels.

### MLCK inhibition with ML-7 diminishes TRPM7 activity

**Figure 4:**
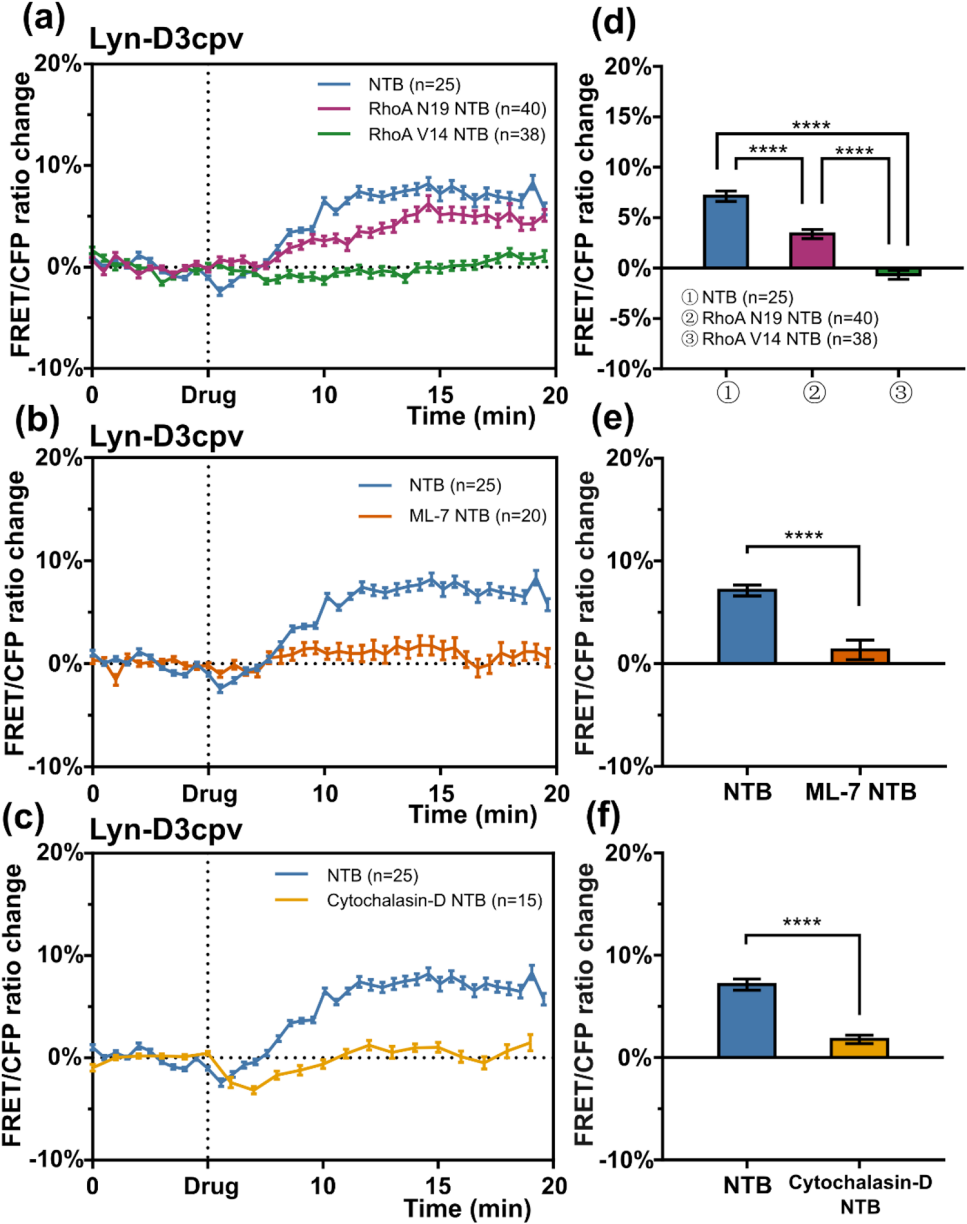
Graphs of the fluorescence resonance energy transfer (FRET)/enhanced cyan fluorescent protein (CFP) ratio change obtained with the Lyn-D3cpv biosensor. (**a**) Mutant RhoA lowered transient receptor potential subfamily M member 7 (TRPM7)-generated Ca^2+^ influx. (**b**) Pretreatment with myosin light chain kinase inhibitor (ML-7) or (**c**) cytochalasin-D drugs halted TRPM7 activity. Bar plots with the values of the FRET/CFP ratio change 12 min after the beginning of the experiments; (**d**) from (**a**) (one-way analysis of variance, ****P < 0.0001); (**e**) from (**b**) (Student’s *t* test, ****P < 0.0001), (**f**) from (**c**) (Student’s *t* test, ****P < 0.0001). NTB, naltriben. n, number of cells. Error bars in the line plots represent the standard error of the mean (*P* < 0.05).

**Figure 5:**
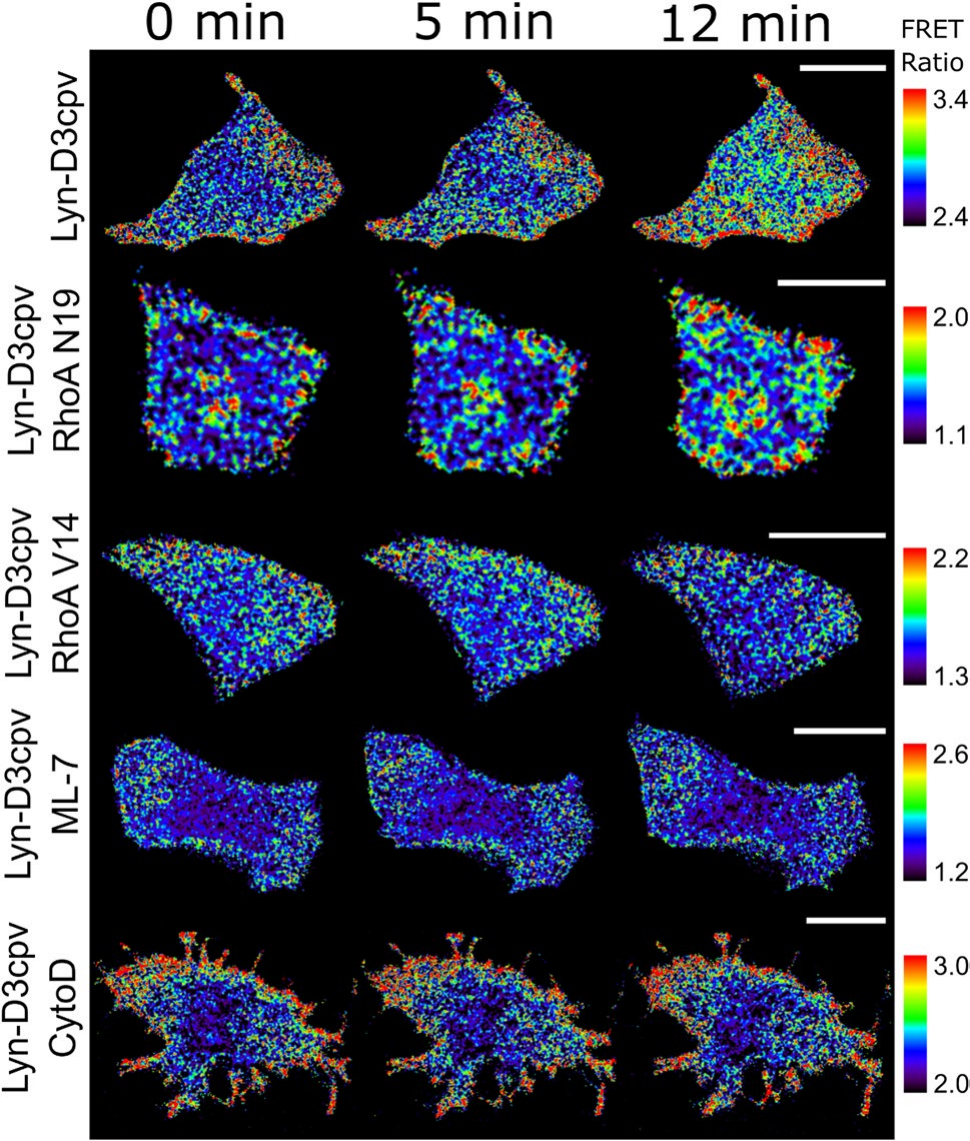
Time-lapse fluorescence resonance energy transfer (FRET) ratio images of the Michigan Cancer Foundation-7 cells in false colors. Naltriben was added 5 min after the beginning of the experiment. The colored scale bar represents the range of the biosensor FRET/enhanced cyan fluorescent protein (CFP) emission ratios. The warmer the color of the pixel, the higher the Ca^2+^ concentration reported. Chemical pretreatment or RhoA mutant vector co-transfection lowered TRPM7-induced Ca^2+^ influx, hence, diminished the FRET/CFP ratio signal change. CytoD, cytochalasin-D. Scale bar = 20 μm.

MLCK phosphorylates myosin II regulatory light chain and, consequently, myosin binds to actin, forming SFs. This process facilitates actomyosin contractility^11^. To investigate how lowered SF-dependent tension impacts on TRPM7 activity, we pretreated MCF-7 cells with the ML-7 drug, which is an MLCK inhibitor. During live-cell imaging, TRPM7-induced Ca^2+^ influx in pretreated cells decreased significantly compared to that in samples that were not pretreated (Figs. 4, 5). These results show that impaired actomyosin contractility negatively affects TRPM7 channel activity.

### RhoA affects TRPM7-induced Ca^2+^ influx

Rho kinases, as with MLCK, can regulate actomyosin contractility. Rho GTPases activate Rho kinase, and the highest expressed Rho GTPase is RhoA^22^. Therefore, we investigated whether RhoA mutants can alter TRPM7 channel activity. RhoA V14 is a constitutively active mutant because it binds to GTP constitutively, and RhoA N19 is a dominant-negative mutant because it has a low affinity to its activators (guanine nucleotide exchange factors)^22^. Plasmids encoding these RhoA variants were co-transfected with Lyn-D3cpv into MCF-7 cells. During live-cell imaging after the administration of naltriben, the FRET signal from cells co-transfected with RhoA N19 decreased slightly, and that from cells co-transfected with RhoA V14 remained at the base level (Figs. 4, 5). These results suggest that RhoA activity contributes to the regulation of TRPM7 activity.

### Cytochalasin-D pretreatment negatively altered the Ca^2+^ flow through TRPM7

To further investigate the effect of actomyosin on TRPM7 activity, we examined how the violation of the actomyosin network structural integrity with mycotoxin cytochalasin-D affects the generated calcium ion flux. Cytochalasin-D breaks actin filaments, disrupts the cytoskeletal network, inhibits polymerization, and induces the depolymerization of actin filaments^23,24^. The cells were treated with cytochalasin-D prior to live-cell imaging. Consequently, the TRPM7 channels showed lower activity in the cells treated with mycotoxin than those that were not treated (Figs. 4, 5). Additionally, these cells changed their morphology, and this result is in accordance with that of a study by Schliwa^24^ (Fig. 5). Thus, the ordered structure of the actomyosin network is important for TRPM7 channel activity.

### Nocodazole pretreatment lowers the response of TRPM7 to naltriben

**Figure 6:**
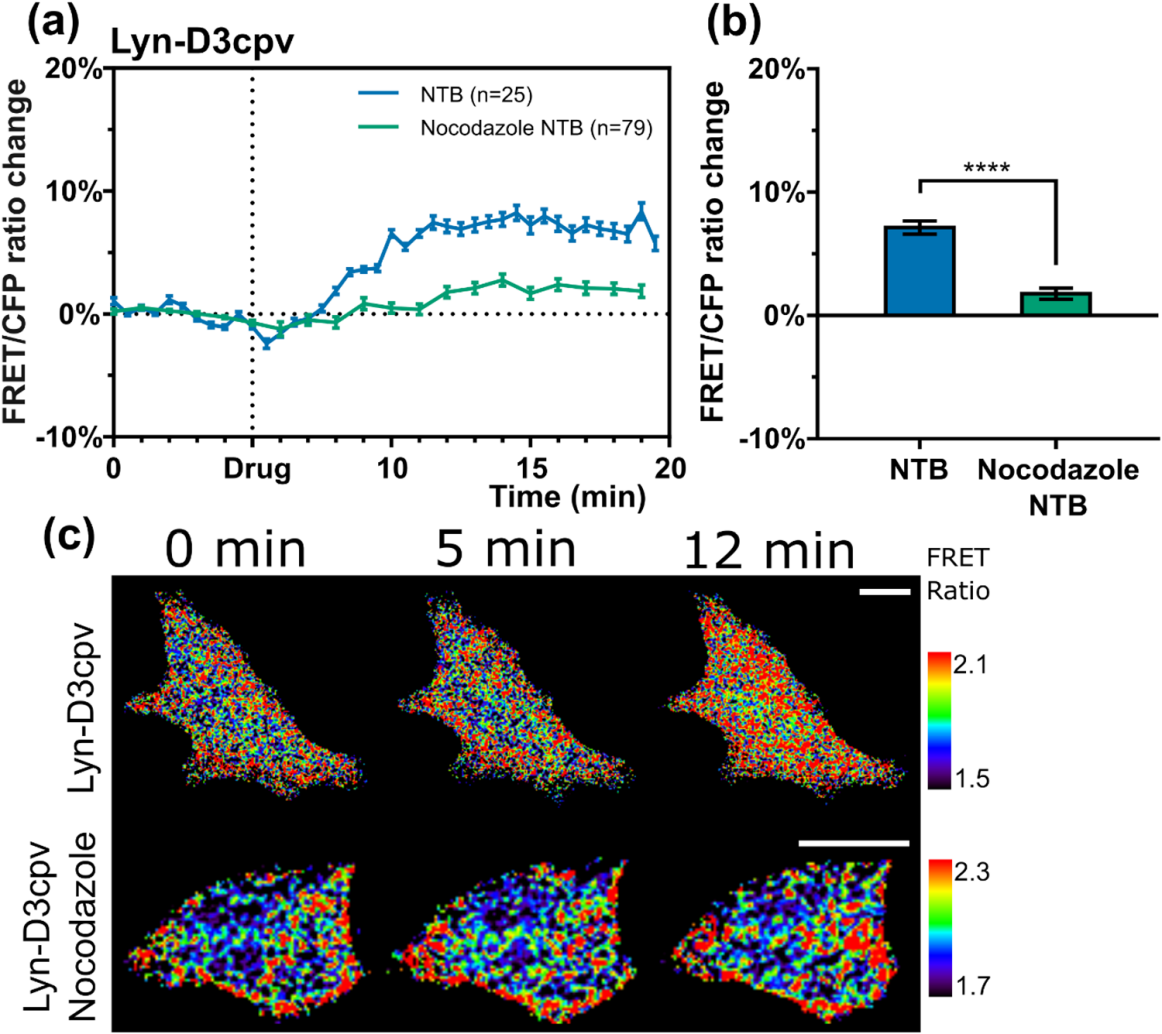
(**a**) Line graphs showing the effect of nocodazole pretreatment on TRPM7 activity. (**b**) Bar chart with the values of the fluorescence resonance energy transfer (FRET)/ enhanced cyan fluorescent protein (CFP) ratio change 12 min after the beginning of the experiment from (**a**). Student’s *t* test, ****P < 0.0001. NTB, naltriben; n, number of cells. Error bars in the line plots represent the standard error of the mean (*P* < 0.05). (**c**) Time-lapse FRET ratio images of the Michigan Cancer Foundation-7 cells in false colors. The colored scale bar represents the range of the biosensor FRET/CFP emission ratios. The warmer the color of the pixel, the higher the Ca^2+^ concentration. Scale bar = 20 μm.

After examining the effect of actomyosin on TRPM7 activity, we investigated whether a similar response may be triggered by changes in another important component of the cytoskeleton; microtubules. Because nocodazole causes the depolymerization of microtubules^32^, we exposed the treatment group cells to it before imaging. Nocodazole-treated cells showed a reduction in TRPM7 activation induced by naltriben compared to the untreated cells (Fig. 6). To further investigate whether the activity of TRPM7 is reversed when microtubules are recovered, we examined TRPM7 activity in response to naltriben after nocodazole washout. As a result, we observed that there was no significant difference in TRPM7-induced Ca^2+^ influx between the control (n = 7) and nocodazole washout group (n = 9), supporting that TRPM7 activity reverts after microtubule repolymerization (Supplementary Fig. 2). Therefore, our data demonstrate that the structural support of microtubules is essential for TRPM7 function.

### FAK inhibition with PF-573228 and Src inhibition with PP1 both depressed TRPM7-induced Ca^2+^ influx

**Figure 7:**
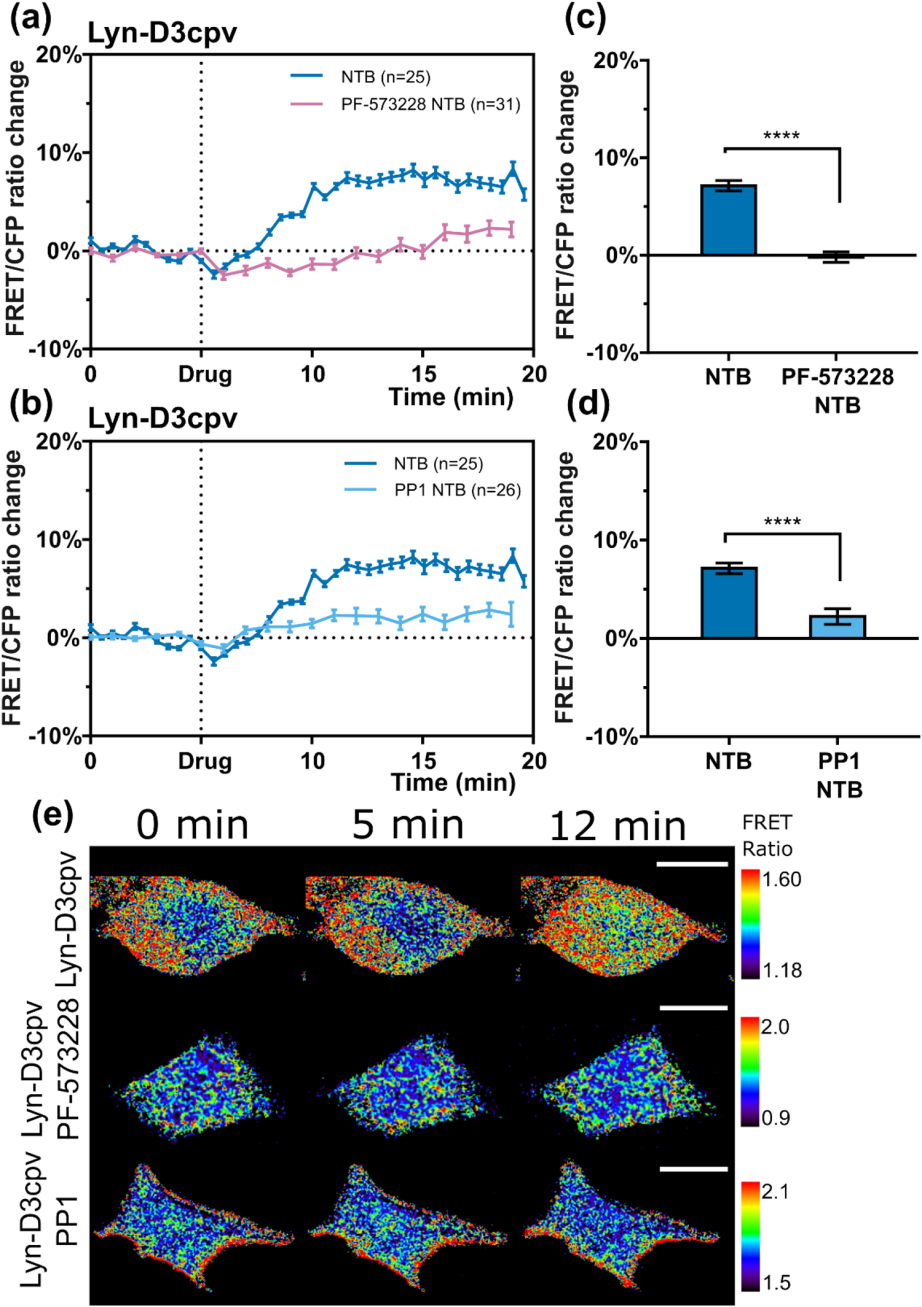
Line graphs showing that (**a**) PF-573228 and (**b**) PP-1 chemical pretreatment of MCF-7 cells decreased TRPM7-induced Ca^2+^ ion influx. The bar chart with the values of fluorescence resonance energy transfer (FRET)/Enhanced cyan fluorescent protein (CFP) ratio change 12 min after the beginning of the experiment; (**c**) from (**a**) and (**d**) from (**b**). Student’s *t* test, ****P < 0.0001. NTB, naltriben; n, number of cells. Error bars in the line plots represent the standard error of the mean (*P* < 0.05). (**e**) Time-lapse FRET ratio images of the MCF-7 in false colors. The colored scale bar represents the range of the biosensor FRET/CFP emission ratios. The warmer the color of the pixel, the higher the Ca^2+^ concentration reported. Scale bar = 20 μm.

FAK is an important structural and functional component of FA and is involved in FA turnover^17^. Reduced FA turnover can affect the organization of lipid rafts^6,8^. Since we identified that TRPM7 is mainly active in areas of lipid rafts, we suggest that a reduced FA turnover affects TRPM7 activity. To test this hypothesis, cells were treated with PF-573228 before imaging. PF-573228 inhibited FAK phosphorylation and turnover^33^. The results showed reduced TRPM7 activity in cells treated with PF-573228 compared to that in untreated cells (Fig. 7). Src-induced FAK phosphorylation regulates both actin and adhesion dynamics^18^. To investigate whether Src influences TRPM7 activity, PP1, an Src inhibitor^34^, was used for cell pretreatment, and live-cell imaging was then performed. The Ca^2+^ influx decreased, and this result was similar to the effect observed in cells treated with PF-573228 (Fig. 7).

## Discussion

In this study, real-time live-cell imaging experiments were conducted, and, as a reporter, the FRET-based biosensor D3cpv and its derivatives were used to investigate the features of TRPM7 channel regulation by the mechanical cues of cells. To reveal how the TRPM7 channels were distributed relative to the PM domains, biosensors constructed prior to this study were used. Lyn-D3cpv, a biosensor designed to target lipid rafts, contains at its N-terminus a sequence from Lyn kinase, which undergoes post-translational acylation in the cells, such as myristoylation and palmitoylation. The residues of saturated and monounsaturated fatty acids attached in this process have a spatial affinity for densely packed lipid rafts. For Kras-D3cpv, the sequence from KRas was attached to the C-terminus of the biosensor and underwent prenylation in the cells. As a result, the attached bulky polyunsaturated chains were pushed into the non-DRM domains of the PM, which are arranged more spaciously. Because mutated calmodulin in the D3cpv construct binds available calcium ions quickly, it is possible to track the change in concentration of calcium ions precisely at the cell PM domains of interest (Fig. 1).

We found that activation of TRPM7 with naltriben in MCF-7 cells occurred mostly for Lyn-D3cpv compared to Kras-D3cpv (Fig. 2). This indicates that TRPM7 is predominantly located at lipid rafts in the cell PM. TRPM7 may be a crucial regulator of Ca^2+^ mobilization at DRM microdomains of the cell PM^19^, which is supported by the results of the present study.

We assessed how the disruption of lipid rafts affects the activity of TRPM7 by pretreating MCF-7 cells with MβCD before live-cell imaging. MβCD captures the cholesterol from lipid rafts, thus, destroying its structure. Live-cell imaging showed that such disruption halts TRPM7 activity at the DRM domains of the PM (Fig. 2). This suggests that the presence of lipid rafts is necessary for TRPM7 activity, which is consistent with the results of a previous study^19^.

We used various approaches to elucidate how altered actomyosin contractility affects TRPM7 channel activity. To influence the contractility of actomyosin by inhibiting MLCK, the cells were pretreated with the ML-7 drug. To study the effect of altered RhoA activity on TRPM7, mutant RhoA constructs were co-transfected with the Lyn-D3cpv biosensor into MCF-7 cells.

Before myosin can bind to actin to form SF filaments, the myosin II regulatory light chain needs to be phosphorylated by MLCK. The inhibition of MLCK lowers SF tension^11^. After pretreatment with ML-7, live-cell imaging showed that the TRPM7-induced Ca^2+^ influx decreased compared to that of the control samples (Fig. 4). This indicates that impaired actomyosin contractility negatively affects TRPM7 channel activity. Additionally, actin filament disruption with cytochalasin-D significantly lowered Ca^2+^ flow through TRPM7, which is in agreement with the aforementioned results (Fig. 4).

RhoA is the most expressed Rho-kinase in cells and can regulate actomyosin contractility but via a different mechanism than that described above. The difference is that, unlike with MLCK, the path through Rho kinases does not depend on Ca^2+^ signaling. RhoA V14 is a constitutively active mutant due to constant GTP binding. Actomyosin is constantly polymerizing under its effect, and a constant SF tension is generated^13,14^. RhoA N19 has a lower affinity with guanine nucleotide exchange factors than that of the wild-type; thus, it functions as a dominant-negative mutant. As a result of the live-cell imaging of cells with co-transfected RhoA mutants, the absence of TRPM7-generated Ca^2+^ influx in cells with constitutively active RhoA V14 mutant was found. RhoA V14 creates excess SF formation, which prevents FA disassembly^7^. In the absence of FA turnover, lipid rafts are not recruited in order around FA^8^; thus, TRPM7 activity is lowered.

For co-transfected RhoA N19 in cells, where SF tension was weakened, the level of TRPM7-induced Ca^2+^ influx decreased considerably (Fig. 4), which was consistent with the results of a previous study of the RhoA regulation effects on Ca^2+^ oscillations^35^. The results of the present study are in accordance with those of a previous study^35^ regarding the essential balanced RhoA/ROCK activity. Therefore, RhoA activity contributes to the regulation of TRPM7 activity.

In both the ML-7 pretreatment and the co-transfected RhoA N19, the SF tension was reduced. Therefore, a similar response from the channel was expected but did not occur. After pretreatment with ML-7, the strength of the signal indicating the influx of calcium ions through the TRPM7 channel was significantly lower than that of RhoA co-transfection. This apparent contradiction may be explained by the different mechanisms of the actomyosin contractility regulation by MLCK and RhoA. In a previous study regarding actomyosin contraction regulation^36^, it was concluded that the Ca^2+^-calmodulin-dependent pathway regulates the rapid contraction of peripheral SFs, and Rho-kinase maintains more finely-tuned SF contraction in cells. Similar conclusions were reached in another study regarding the membrane protrusions during fibroblast migration^37^. ML-7 rapidly changes the contraction of SFs at the cell periphery and may rupture lipid rafts around the FAs. It is possible that due to the violation of the optimal SF tension with ML-7 (or with cytochalasin-D SF destruction), the normal turnover of FA is hampered, which leads to the decreased activity of TRPM7. This may explain the lower level of TRPM7-induced Ca^2+^ influx signal in the presence of ML-7 compared to that in the presence of RhoA N19. As in the studies discussed above, nocodazole treatment resulted in decreased TRPM7 activity. With the destruction of microtubules, FA turnover was hindered^7^ and the recruitment of lipid rafts to nascent FA decreased, to which the decreased activity of TRPM7 was related.

The hypothesis that the reduced FA turnover affects the TRPM7 activity was confirmed by the addition of PF-573228 or PP1, significantly reducing TRPM7 activity. FAK phosphorylation by Src is important for FA turnover^18^; thus, inhibition of these kinases leads to the reduced recruitment of lipid rafts to FA and decreased TRPM7 activity. Additionally, TRPM7 not only localizes together with the calcium-dependent protease m-calpain near FA but also regulates m-calpain^38^. Without TRPM7 channel activity, m-calpain does not subject FAK to proteolysis, which leads to the reduced FA turnover and decreased recruitment of lipid rafts to FAs. This study confirms co-location of TRPM7 and peripheral focal adhesions, and the excessive activity of TRPM7 leads to cell rounding. As such, TRPM7-medicated Ca^2+^ fluxes might be implicated in actomyosin remodeling and subsequent cell adhesion and migration by mediating focal adhesion complexes^4,41,42^. In addition, in the absence of a local influx of calcium ions, the activity of Ca^2+^-calmodulin-dependent MLCK should decrease. This should further reduce tension in the actomyosin network. Taken together, these processes should also lead to decreased TRPM7 activity, looping the sequence.

**Figure 8:**
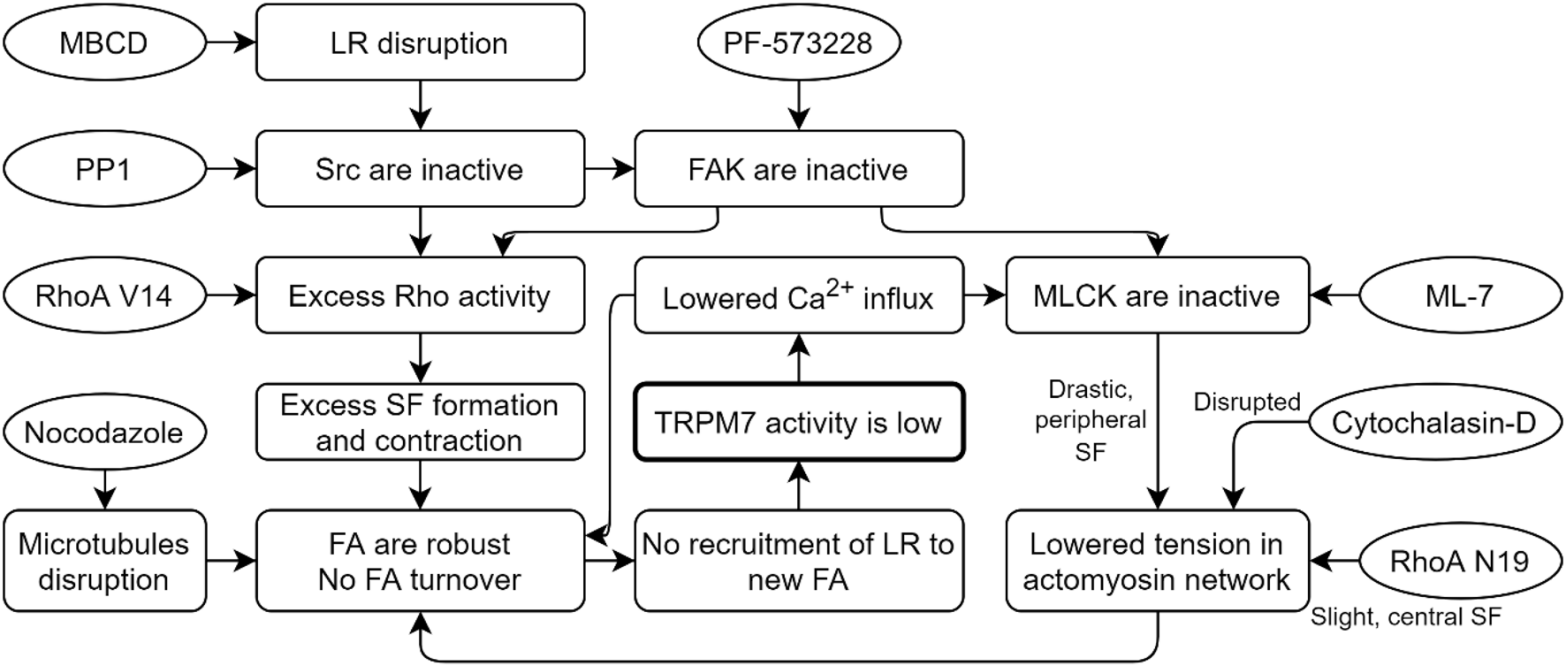
Flowchart showing the regulation of TRPM7 activity by the cell mechanosensitive apparatus components. Lipid raft disruption leads to Src inactivity^39^. Inactive Src does not phosphorylate FAK^17^ and allows RhoA to be excessively active, which leads to excess SF formation and contraction, which causes FA robustness and hampers turnover^7^. The destruction of microtubules has the same effect on FA turnover^16^. FAK influences both Rho and MLCK^40^. Inactive MLCK drastically lowers SF tension on the periphery^36^, which may interfere with FA turnover. All interventions in this system lowered TRPM7 activity. Restricted Ca^2+^ influx affected Ca^2+^-calmodulin-dependent MLCK^11^ and FA turnover^38^.

In conclusion, in this study, we found that TRPM7 activity is dependent on the mechanical cues of the cell. TRPM7 activity was mainly detected at lipid rafts. In all cases of intervention in the considered system, the flow of calcium ions through the TRPM7 channel decreased. These results lay a foundation for the understanding of mechanosensitive channel regulation mechanisms and indicate that TRPM7 is part of a self-regulating mechanosensitive system (Fig. 8), carrying out constant adjustments for effective adaptation to extracellular conditions, which is necessary for cell survival.

## Supplementary Information

Supplementary Figures.

## Abbreviations

D3cpv: Design 3 with circularly permuted venus
DAPI: 4′,6-Diamidino-2-phenylindole
DMEM: Dulbecco’s modified Eagle’s medium
DRM: Detergent-resistant membrane
ECFP: Enhanced cyan fluorescent protein
FA: Focal adhesions
FAK: Focal adhesion kinase
FBS: Fetal bovine serum
FITC: Fluorescein isothiocyanate
FRET: Fluorescence resonance energy transfer
GTPase: Guanosine triphosphatase
IF: Immunofluorescence
MβCD: Methyl-beta-cyclodextrin
mCam: Mutated calmodulin
MCF-7: Michigan cancer foundation-7
ML-7: Myosin light chain kinase inhibitor
MLCK: Myosin light chain kinase
m-smMLCKp: Mutated smooth muscle myosin light chain kinase peptide
P/S: Penicillin–streptomycin
PBS: Phosphate-buffered saline
PM: Plasma membrane
ROCK: Rho-associated coiled-coil forming kinase
SF: Stress fiber
