# Supplementary material for: Distinct calcium regulation of TRPM7 mechanosensitive channels at plasma membrane microdomains visualized by FRET-based single cell imaging
Source: Sci Rep. 2021 Sep 9;11:17893. doi: 10.1038/s41598-021-97326-z (PMC8429465; doi:10.1038/s41598-021-97326-z)
Supplement: Supplementary file 1 — Supplementary Figures. [file 41598_2021_97326_MOESM1_ESM.docx]

Supplementary Information

**Distinct Calcium Regulation of TRPM7 Mechanosensitive Channels at Plasma Membrane Microdomains Visualized by FRET-based Single Cell Imaging**

Irina Starostina ^1#^, Yoon-Kwan Jang ^1#^, Heon-Su Kim ^1^, Jung-Soo Suh ^1^, Sang-Hyun Ahn ^1^, Gyu-Ho Choi ^1,2^, Myungeun Suk^3,*^, and Tae-Jin Kim^1,2,*^

^1^Department of Integrated Biological Science, Pusan National University, Pusan 46241, Republic of Korea, ^2^Department of Biological Sciences, Pusan National University, Pusan 46241, Republic of Korea, ^3^Department of Mechanical Engineering, Dong-Eui University, Busan 47340, Republic of Korea

^#^ These authors contributed equally to this work.


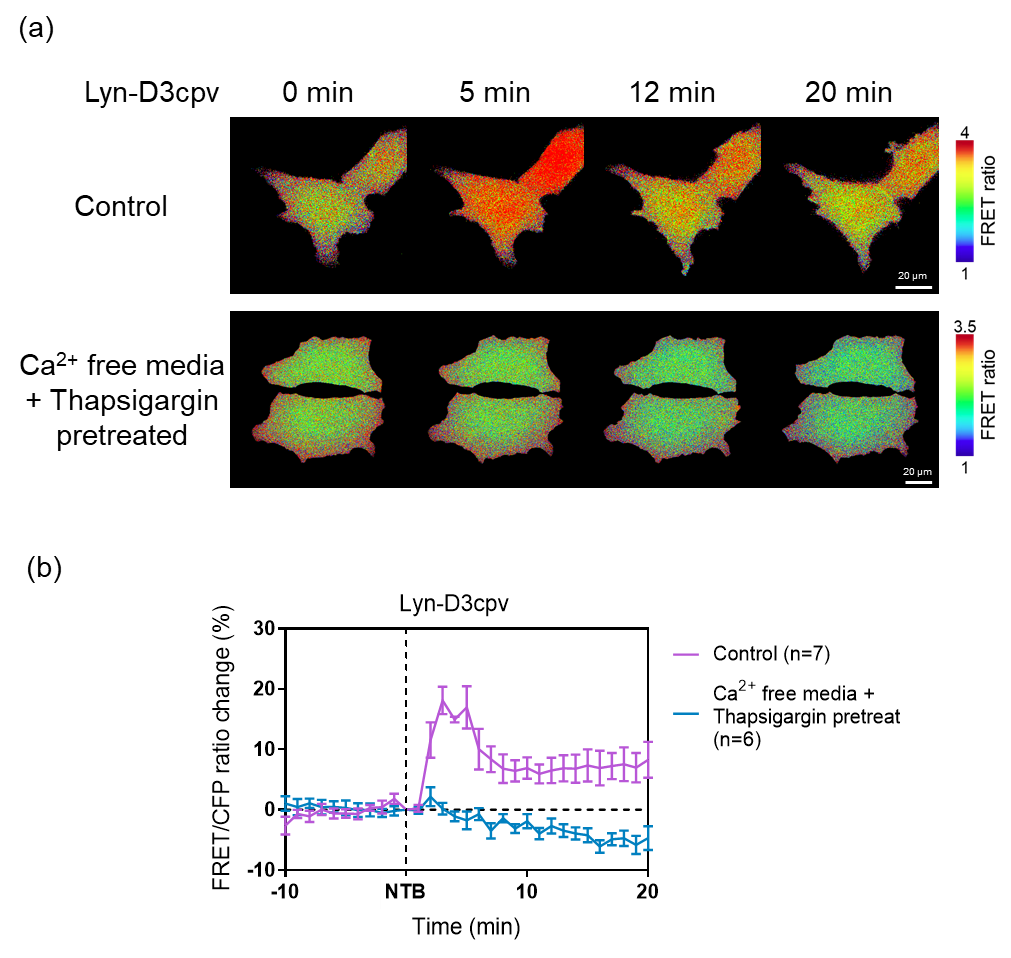


Supplementary Figure 1. (a) Time-lapse FRET ratio images of the MCF-7 cells in false colors. The colored scale bar represents the range of the biosensors’ FRET/CFP emission ratios. The warmer the color of the pixel, the higher the Ca^2+^ concentration reported. Scale bar = 20 μm. (b) Line graph of FRET/CFP ratio change obtained with Lyn-D3cpv biosensor. NTB = Naltriben (50 μM); n = number of the cells.


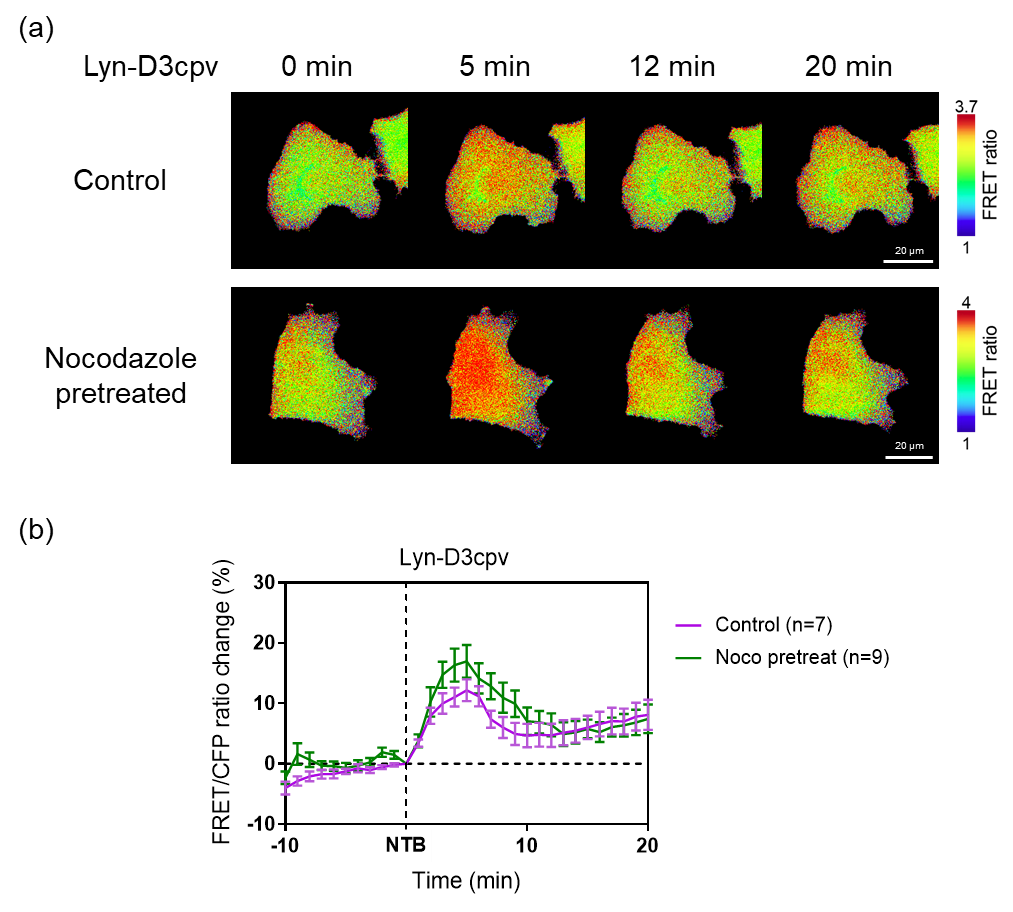


Supplementary Figure 2. (a) Time-lapse FRET ratio images of the MCF-7 cells in false colors. The colored scale bar represents the range of the biosensors’ FRET/CFP emission ratios. The warmer the color of the pixel, the higher the Ca^2+^ concentration reported. Scale bar = 20 μm. (b) Line graph of FRET/CFP ratio change obtained with Lyn-D3cpv biosensor. NTB = Naltriben (50 μM); n = number of the cells.
